# Diagnostic features of quantitative comb-push shear elastography for breast lesion differentiation

**DOI:** 10.1371/journal.pone.0172801

**Published:** 2017-03-03

**Authors:** Mahdi Bayat, Max Denis, Adriana Gregory, Mohammad Mehrmohammadi, Viksit Kumar, Duane Meixner, Robert T. Fazzio, Mostafa Fatemi, Azra Alizad

**Affiliations:** 1 Department of Physiology and Biomedical Engineering, Mayo Clinic College of Medicine, Rochester, MN, United States of America; 2 Department of Radiology, Mayo Clinic College of Medicine, SW, Rochester, MN, United States of America; 3 Department of Internal Medicine, Mayo Clinic College of Medicine, SW, Rochester, MN, United States of America; University of South Alabama Mitchell Cancer Institute, UNITED STATES

## Abstract

**Background:**

Lesion stiffness measured by shear wave elastography has shown to effectively separate benign from malignant breast masses. The aim of this study was to evaluate different aspects of Comb-push Ultrasound Shear Elastography (CUSE) performance in differentiating breast masses.

**Methods:**

With written signed informed consent, this HIPAA- compliant, IRB approved prospective study included patients from April 2014 through August 2016 with breast masses identified on conventional imaging. Data from 223 patients (19–85 years, mean 59.93±14.96 years) with 227 suspicious breast masses identifiable by ultrasound (mean size 1.83±2.45cm) were analyzed. CUSE was performed on all patients. Three regions of interest (ROI), 3 mm in diameter each, were selected inside the lesion on the B-mode ultrasound which also appeared in the corresponding shear wave map. Lesion elasticity values were measured in terms of the Young’s modulus. In correlation to pathology results, statistical analyses were performed.

**Results:**

Pathology revealed 108 lesions as malignant and 115 lesions as benign. Additionally, 4 lesions (BI-RADS 2 and 3) were considered benign and were not biopsied. Average lesion stiffness measured by CUSE resulted in 84.26% sensitivity (91 of 108), 89.92% specificity (107 of 119), 85.6% positive predictive value, 89% negative predictive value and 0.91 area under the curve (*P<*0.0001). Stiffness maps showed spatial continuity such that maximum and average elasticity did not have significantly different results (*P* > 0.21).

**Conclusion:**

CUSE was able to distinguish between benign and malignant breast masses with high sensitivity and specificity. Continuity of stiffness maps allowed for choosing multiple quantification ROIs which covered large areas of lesions and resulted in similar diagnostic performance based on average and maximum elasticity. The overall results of this study, highlights the clinical value of CUSE in differentiation of breast masses based on their stiffness.

## Introduction

Ultrasonography (US) is a commonly used diagnostic tool for palpable or mammographically detected breast masses [1]; however, conventional ultrasound suffers from low specificity [2] resulting in large number of unnecessary benign biopsies [3]. It is well known that benign breast masses are more often stiffer than normal breast tissue but softer than malignant masses [4]. To improve lesion differentiation, elastography techniques have emerged to help provide a noninvasive assessment of pathology based on mechanical properties. Strain elastography is one such technique that measures tissue strain or tissue displacement in response to internally induced motions as well as external quasi-static compression. The clinical value of this technique has been shown in the study of breast lesions [5, 6]. However, operator dependency and challenges in quantifying elasticity continue to be the main shortcomings of the strain elastography. Emerging shear wave elastography (SWE) techniques may overcome these problems. These techniques use acoustic radiation force (ARF) to generate shear waves and tissue elasticity can be quantified by measuring shear wave speeds [7, 8]. Since shear wave speed increases with tissue stiffening, its estimation by shear wave elastography can help characterize and differentiate benign from malignant breast masses. SWE techniques based on point shear wave elastography (p-SWE) as described in [9] as well as shear wave elastography of an area of interest have been used for characterization of breast masses [10–16].

Comb-push Ultrasound Shear Elastography (CUSE) is a recently developed technique that uses multiple simultaneous laterally spaced ARF beams to generate a full field of view (FOV) with shear waves travelling in both lateral directions [17, 18]. This type of excitation enhances the continuity of the reconstructed elasticity maps covering the entire lesion area as seen in the B-mode image [18]. This continuity, in turn, simplifies assessment and interpretation of the lesion stiffness [19–21].

In this study, we evaluate the performance of comb-push shear elastography implemented on a clinical ultrasound machine in the differentiation of breast masses by correlating the quantitative values of lesion elasticity, with pathology as the reference standard.

## Materials and methods

This study was approved by the Mayo Clinic institutional review board (IRB Application #12–003329) and was Health Insurance Portability and Accountability Act (HIPAA) compliant. A written informed consent was obtained from each participant.

The aim of this prospective study was to evaluate the performance of comb-push ultrasound shear elastography (CUSE) in differentiating malignant from benign lesion in a group of pre-biopsy patients. A GE LOGIQ E9 (LE9) machine with CUSE capability was used for both usual B-mode scanning as well as acquiring SWE data.

### Study population

From April 2014 through August 2016, 226 consecutive female patients (>18 years old) with suspicious breast lesions identified by breast physical examination and/or imaging studies and scheduled for US guided biopsy were enrolled in this study. No patients had breast implants or history of mastectomy. Three patients were excluded from the patient population pool due to difficulties in acquiring elasticity images. A total of 227 breast masses from the remaining 223 patients were examined (four patients had 2 lesions each). Breast masses characterized as BI-RADS (Breast Imaging Reporting and Data System) category 2 (1 patient; 1 lesion), 3 (11 patients; 11 lesions), 4 (158 patients; 161 lesions) and 5 (53 patients; 54 lesions) after conventional clinical ultrasound were included. Three BI-RADS 3 and one BI-RADS 2 patients did not undergo biopsy and had stable lesions for more than a year with 6-month follow-up appointments. The 8 remaining BI-RADS 3 patients requested or were recommended to have a biopsy (i.e., due to changes in lesion size) during follow-up. The mean patient age was 54.93±14.96 years, and the range was 19–85 years. The results of shear wave elastography were correlated with those of pathology and statistically analyzed.

### US imaging and shear wave elastography

An expert sonographer with 28 years of experience in breast US conducted the US examinations, according to the American Institute of Ultrasound in Medicine practice guidelines for performing breast US [22]. Breast lesions were localized using the B-mode scanning, and then SWE data were acquired prior to biopsy. After standard conventional US, SWE examinations were performed by our experienced post-doctoral fellows. Shear wave elastography was performed by GE Logiq E9 clinical scanner equipped with the CUSE capability [18] using a 9L linear array probe with 2–8 MHz frequency range (GE Healthcare, Wauwatosa, WI). A rectangle-shaped field of view (FOV) was set for SWE acquisition, and stiffness was displayed as a color map in that FOV. In addition to the lesion, the FOV also included normal breast tissue adjacent to the lesion to visualize the stiffness contrast. SWE measurements were acquired while minimizing the pre-compression as well as instructing the patients to suspend respiration during the data acquisition (approximately 3 seconds). A corresponding B-mode image was also displayed and used to delineate the margins of each mass. Three non-overlapping 3mm regions of interest (ROIs) were selected from inside the lesion on the B-mode image using a dual panel measurement tool available on the scanner. This feature allowed for selecting simultaneous circular ROI on the B-mode image while measuring the values from the shear wave speed map. The number of 3mm ROI was reduced for lesions with a size less than 9 mm in one dimension or less than 6mm in two perpendicular dimensions. The stiffness of the surrounding normal tissue was also measured using a 3mm ROI outside the lesion site. The maximum shear wave speed (*V*_*max*_)_,_ mean shear wave speed (*V*_*mean*_), minimum shear wave speed (*V*_*min*_), and shear wave speed standard deviation (*V*_*SD*_) were automatically calculated by the US system. The shear wave speed estimates were then converted to Young’s modulus, *E*_mean_, *E*_max_ and *E*_SD_, assuming a tissue density of 1000 kg/m^3^ [20].

All de-identified SWE and conventional US images were securely stored on the US system’s internal hard disk for subsequent offline statistical analysis.

### Histopathological examination

Histopathological results were available for all patients and were used as the reference standard. Specifically, all patients underwent US guided core needle biopsy or surgical excision biopsy as the part of their clinical care (except for 5 probably benign cases) subsequent to conventional diagnostic imaging and completion of our research protocol examination. A 14-gauge needle (Achieve biopsy device, CareFusion Corporation, Waukegan, IL) was used by one of our board-certified radiologists to obtain five core biopsy samples for each case. Histopathological diagnosis was made by an experienced pathologist with more than 15 years of experience. The results of surgical pathology were the same of core biopsies in the cases that surgical pathology was performed (malignant cases).

### Statistical analysis

Statistical analyses were performed by using MedCalc (MedCalc Software bvba Ver. 15.8, Belgium). The mean, maximum and normalized mean and normalized maximum elasticity values calculated from three ROIs were reported as the final estimates for each lesion. The elasticity values were correlated to the results of pathology in terms of a receiver operator curve (ROC). Sensitivity, specificity, positive predictive values (PPV) and Negative predictive Values (NPV) were calculated. The prevalence rate was 41.5% based on the meta-analysis study performed in [23]. Both point estimates and 95% confidence intervals (CIs) were used. Optimal thresholds were determined to have an acceptable balance on sensitivity and specificity. A two-sided Mann-Whitney rank-sum test (*P <* 0.05) was used to compare the quantitative SWE values for the differentiation.

## Results

### Participant demographics and lesion

Two hundred twenty seven masses in 223 patients were analyzed. Biopsy was performed in all BI-RADS 4 and 5 breast lesions. Four BI-RADS 3 and one BI-RADS 2 cases were considered probably benign and therefore were not biopsied. Overall, 47.6% (108/227) of lesions were malignant, and 52.4% (119/227) were benign (Table 1).

**Table 1:** Summary of classification performance for different elasticity measures.

	*E*_*mean*_	*E*_*mean*, *1-ROI*_	*E*_*max*_
**Sensitivity**	• 84.26% • CI: 76.0%-90.6% • 91/108	• 92.59% • CI: 85.9%-96.7% • 100/108	• 87.96% • CI: 80.3%-93.4% • 95/108
**Specificity**	• 89.92% • CI: 83.0% -94.7% • 107/119	• 77.31% • CI: 68.7%-84.5% • 92/119	• 88.24% • CI: 81.0%-93.4% • 105/119
**PPV**	• 85.6% • CI: 76.7%-92.0% • Prevalence: 41.5%	• 74.3% • CI: 65.4%-81.9% • Prevalence: 41.5%	• 84.1% • CI: 75.4%- 90.7% • Prevalence: 41.5%
**NPV**	• 89.0% • CI: 82.4%-93.7% • Prevalence: 41.5%	• 93.6% • CI: 87.3%-97.4% • Prevalence: 41.5%	• 91.2% • CI: 84.9%-95.5% • Prevalence: 41.5%
**Optimal Cut off**	• 61.93kPa	• 42.42kPa	• 105.74 kPa
**Area under the curve**	• 0.906 • CI: 0.860–0.940	• 0.885 • CI: 0.837–0.924	• 0.892 • CI: 0.844–0.929

### Diagnostic performances of CUSE

**Fig 1:**
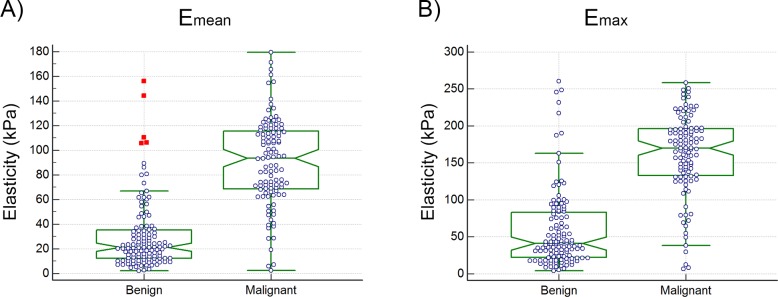
**(A)** Elasticity distribution based on mean elasticity values and **(B)** Elasticity distribution based on maximum elasticity values for benign and malignant masses. The red markers show the data points outside the upper quartile plus 3 times the interquartile range. Median separation was 71.65kPa for mean elasticity and 133.27kPa for maximum elasticity.

Maximum elasticity, *E*_*max*_, and mean elasticity, *E*_*mean*_, were significantly higher in malignant breast lesions than in benign lesions, (*P* < 0.0001 for all). The mean Young’s moduli for benign and malignant lesions were found to be 30.18±27.81kPa and 90.66±35.55kPa, respectively. The maximum Young’s moduli were 59.20±53.88kPa and 161.64±55.03kPa for benign and malignant masses, respectively. The notch plots of the elasticity values for the benign and malignant cases along with the data points for the mean and maximum elasticity are shown in Fig 1A and 1B.

**Fig 2:**
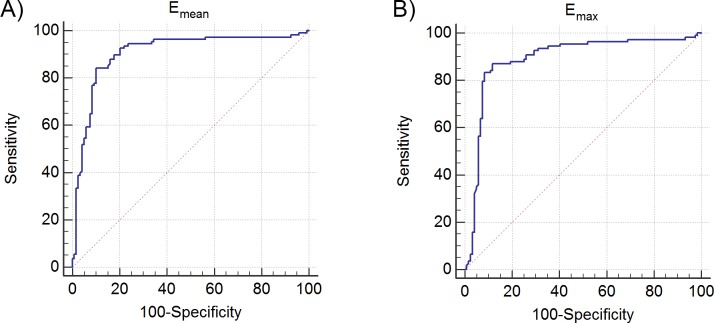
**(A)** ROC curve based on mean elasticity values and **(B)** ROC curve based on maximum elasticity values.

ROC analysis of mean (max) elasticity revealed optimum thresholds of 61.93kPa (105.74kPa) (Fig 2A and 2B). The mean elasticity resulted in 84.26% (91/108, CI: 76%-90.6%) sensitivity, 89.92% (107/119, CI: 83%-94.7%) specificity, 85.6% (CI: 76.7%-92.0%) positive predictive value, 89.0% (CI: 82.4%-93.7%) negative predictive value and an area under the curve of 0.906 (CI: 0.860–0.940). The maximum elasticity resulted in 87.96% (95/108, CI: 80.3%-93.4%) sensitivity, 88.24% (105/119, CI: 81.0%-93.4%) specificity, 84.1% (CI: 75.4%-90.7%) positive predictive value, 91.2% (CI: 84.9%-95.5%) negative predictive value and an area under the curve of 0.892 (CI: 0.844–0.929).

The detection accuracy based on maximum elasticity (*E*_*max*_) was not found to be significantly different from that of average elasticity (*E*_*mean*_) (*P* > 0.21, area under the curve difference < 0.0172).

The average elasticity from a single 3mm ROI, *E*_*mean*, *1-ROI*_, resulted in 92.59% (CI: 85.9%-96.7%) sensitivity, 77.31% (CI: 68.7%-84.5%) specificity, 74.3% (CI: 65.4%-81.9%) positive predictive value, 93.6% (CI: 87.3%-97.4%) negative predictive value and an area under the curve of 0.885 (CI: 0.837–0.924) using an optimal cut-off value of 42.42 kPa.

A summary of the statistical analysis is shown in Table 1. Misclassified cases based on average elasticity are summarized in Table 2.

**Table 2:** False positive cases based on mean elasticity.

False Positive cases (12 cases)	*E*_*mean*_(kPa)	*E*_*mean*, *1-ROI*_(kPa)	*E*_*max*_(kPa)	BI-RADS (US)
Intraductal papilloma with associated apocrine cyst.	155.95	187.70	231.53	4
Fibroadenomatoid nodule	144.07	144.07	217.26	4
Complex sclerosing lesion with radial scar, intraductal papilloma	110.41	130.68	151.23	5
Benign breast tissue with fat necrosis	106.21	106.21	190.08	4
Diabetic mastopathy	105.61	113.47	197.80	4
Fat Necrosis	89.43	104.08	248.43	5
Dense stromal fibrosis and foreign body type giant cell reaction, consistent with prior biopsy site and fat necrosis.	86.19	124.42	260.31	4
Fat necrosis with dystrophic calcifications	80.70	141.18	163.10	4
Benign-Fibroadenoma	80.08	83.32	122.50	3
Benign-Intraductal papilloma. Fibrocystic changes including fibroadenomatoid nodule.	73.41	89.43	125.58	4
Intraductal papilloma with usual ductal hyperplasia, apocrine metaplasia and columnar cell change	66.98	74.10	120.40	4
Dense stromal fibrosis with calcifications in benign ducts	65.33	75.00	187.23	4

### Representative cases

**Fig 3:**
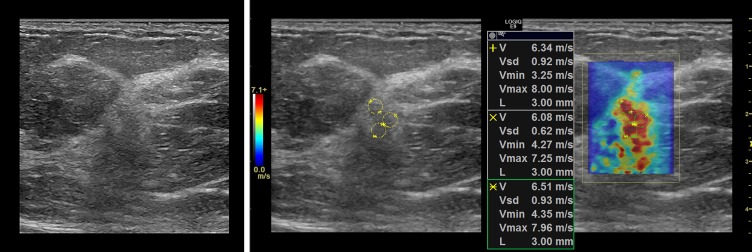
Shear wave map of a 1.4cm malignant mass diagnosed as invasive ductal carcinoma, grade III. *E*_*mean*_ = 117.8kPa.

**Fig 4:**
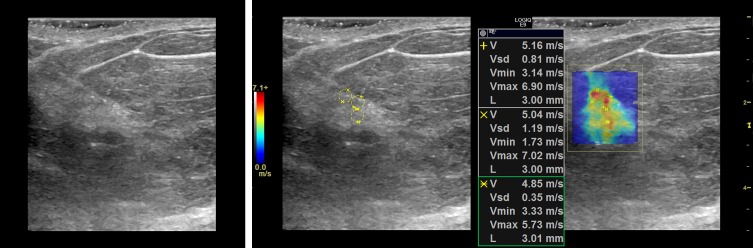
Shear wave speed map of a malignant breast mass diagnosed as invasive ductal carcinoma grade II. *E*_*mean*_ = 76kPa. The lesion contrast on the B-mode image is considerably low due to minimal probe compression used for SWE data acquisition.

**Fig 5:**
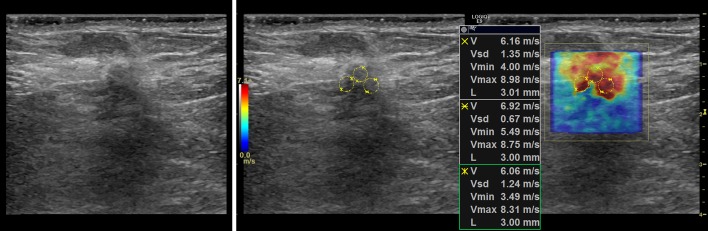
Shear wave map of a 1.05cm malignant breast diagnosed as invasive ductal carcinoma grade I. The average elasticity was *E*_*mean*_ = 120.3 kPa.

**Fig 6:**
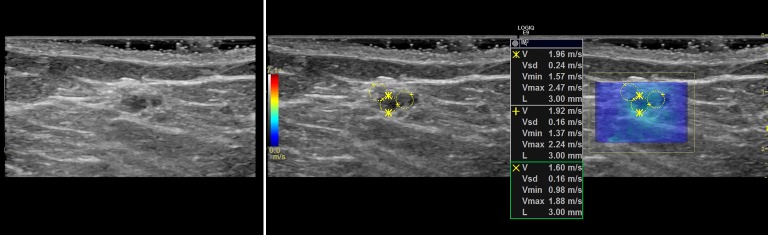
Shear wave speed map of a benign breast mass diagnosed as papilloma. *E*_*mean*_ = 10kPa.

**Fig 7:**
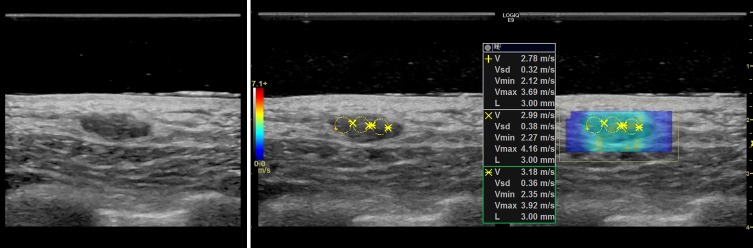
Shear wave speed map of a benign breast mass diagnosed as fibroadenoma. *E*_*mean*_ = 26.7kPa.

In this section, we present the two dimensional shear wave speed maps of five patients with breast lesions using CUSE. The yellow circles on the shear wave images (Figs 3–7) are the ROIs used for shear wave speed calculation.

#### Case 1

The patient was a 62 y/o woman with a breast mass measuring 1.4cm in greatest dimension. Fig 3 shows the B-mode ultrasound and the shear wave speed map of the lesion. Percutaneous core needle biopsy revealed invasive ductal carcinoma (IDC), grade III. The average of the corresponding three 3mm ROI resulted in a mean elasticity value of *E*_*mean*_ = 117.8kPa.

#### Case 2

The patient was a 72 y/o woman with a subareolar mass identified in screening mammography. Conventional B-mode ultrasound demonstrated a 9mm hyperechoic mass with an irregular margin. Fig 4 shows the lesion which was diagnosed as invasive ductal carcinoma grade II after core needle biopsy. In contrast to the previous case, the B-mode image does not present significant contrast on the lesion site. The shear wave speed map, however, presents a continuous stiffness map with surrounding soft boundaries representing normal breast tissue. Compared to the previous case, this lesion shows slightly lower elasticity (*E*_*mean*_ = 76kPa) which can be suggestive of the lower grade IDC [24].

#### Case 3

The patient was an 81 y/o woman with a 1.05cm irregular and hypoechoic breast mass with posterior acoustic shadowing. The B-mode US and shear wave speed map obtained during the SWE study can be seen in Fig 5, where the average elasticity was 120.3kPa. Histopathology analysis revealed this mass to represent invasive ductal carcinoma grade I, measuring 0.3cm in greatest linear extent. The background breast showed lobular carcinoma in situ and atypical lobular hyperplasia, focally involving a radial scar. Although the elasticity was high in this low grade case, studies have reported an increase of stiffness in radial scars [25].

#### Case 4

The patient was a 62 y/o woman with a complex cystic and solid mass that measured 9mm in greatest dimension. Fig 6 presents the B-mode and shear wave map of the lesion diagnosed as benign papilloma following percutaneous core needle biopsy. B-mode ultrasound demonstrates a hypoechoic region with no significant posterior shadowing. The corresponding shear wave speed map appears with no significant contrast indicating a soft lesion. The average elasticity was *E*_*mean*_ = 10kPa.

#### Case 5

The patient was a 45 y/o woman who presented with palpable findings in her right breast, corresponding to an oval circumscribed hypoechoic mass seen with diagnostic ultrasound. Fig 7 (left-side) shows the B-mode image of the mass. A stand-off gel pad was used since the lesion was superficial. The shear speed map indicates a slightly elevated stiffness at the lesion site with an average elasticity of *E*_*mean*_ = 26.7kPa. Biopsy was performed revealing a benign fibroadenoma.

## Discussion

A major advantage of the shear wave elastogrpahy using CUSE is the deep penetration of the radiation force in a large field of view in one acquisition. This type of shear wave excitation was shown to create displacements even in very stiff materials, which can in turn result in more continuous elasticity maps [18]. Two studies [11, 26] highlight considering a stiff rim sign in the differentiation of stiff breast masses where elasticity values are mostly observed on the exterior boundary of the lesion site. The study by Barr [27] also provides insights about the role of weak shear waves within the stiff lesions in misinterpretation of the actual lesion stiffness. However, most of our cases resulted in continuous color coded shear wave velocity maps that covered the entire mass footprint seen in the synchronized B-mode image. These features facilitated ROI selection and aided in interpretation of the elasticity maps. In a few cases with posterior B-mode shadowing, the high speed values were extended far below the lesion area which can be attributed to the low signal-to-noise (SNR) of the ultrasound echoes used for tracking shear waves [28].

In comparison to previous reports where elasticity measurement were usually taken from a very small ROI (usually 2mm) and from around the lesion [25, 29], the use of three 3mm ROIs increased the area from which the elasticity measurement were calculated. In addition, multiple smaller ROIs provided the flexibility to cover a large elasticity measurement area in a wide range of lesion geometries as they can be arranged in different directions. This type of ROI selection especially helped in lesions with irregular B-mode boundaries where a single large round or square ROI did not fit. Compared to a single 3mm ROI, the three 3mm ROI criterion resulted in better differentiation statistics (Table 2). One justification for this is that the three ROIs can probably better capture the stiffness heterogeneity of the breast masses than a single 3mm ROI.

The mean elasticity values measured in our study provided a high specificity (89.92%) in pre-biopsy patients (mostly BI-RADS 4 and 5), while maintaining a high sensitivity (84.26%). The diagnostic performance of the maximum elasticity was not found to be significantly different from that of mean elasticity (*P* > 0.21) where the difference between the area under curves of the two measures was negligible (0.0172). This in turn, highlights the uniformity of the estimated elasticity maps. In the case of mean elasticity, false positive cases included: papillomas, complex sclreosing and radial scar lesions, fat necrosis, diabetic mastopathy, and stromal fibrosis with calcifications. Radial scaring also appeared to increase elasticity in some low grade malignant cases (e.g. Case 3 in the results section) which has been reported in previous studies [25].

Each of these examples have been shown to display ultrasound features similar to malignant masses [14, 25, 30]. Presence of calcification in one of four false positive lesions may have contributed to this error. A recent study confirms that the presence of calcification within a lesion can emulate the appearance of high stiffness region when measured by shear wave elasticity techniques [28, 31].

A limitation of our study is that we performed our shear wave technique on patients who were scheduled for breast biopsy, with pathology as gold standard; therefore patients with BI-RADS 4 and 5 category lesions were predominantly selected for evaluation (only one BI-RADS 2 and three BI-RADS 3 were evaluated). For this reason, we could not establish statistical analysis comparison between our technique and B-mode ultrasound alone. Nevertheless, the study by Berg *et*. *al* [32] which was performed in a more comprehensive pool of patients confirms the poor specificity (34%) of B-mode ultrasound alone. A study with higher number of patients with low BIRADS values can help understand the true value of CUSE in differentiation of benign lesions from malignant.

In summary, the overall results of this study prove the high accuracy and reliability of the comb-push shear elastography in the differentiation of breast masses which might have clinical implications in terms of reducing unnecessary biopsies.
